# Biosimilar in Breast Cancer: A Narrative Review

**DOI:** 10.7759/cureus.52828

**Published:** 2024-01-23

**Authors:** Diplina Barman, Tibar Bandyopadhyay, Rounik Talukdar

**Affiliations:** 1 Epidemiology, Indian Council of Medical Research-National Institute of Cholera and Enteric Diseases (ICMR-NICED), Kolkata, IND; 2 Plastic and Reconstructive Surgery, Institute of Post-Graduate Medical Education and Research, Seth Sukhlal Karnani Memorial Hospital, Kolkata, IND

**Keywords:** biosimilar of trastuzumab, cancer therapeutic, monoclonal antibody therapy, breast cancer research, biosimilar

## Abstract

Breast cancer (BC) has been identified as a major public health cancer as it topped the list of most prevalent cancers among women in the last three years. Rigorous research has been conducted to improve the prognosis of cancer therapies since the time of inception. Recent advancements in cancer therapy have introduced monoclonal biosimilars as a promising treatment alternative. Monoclonal antibodies (mAbs), produced through cloning, have demonstrated effectiveness in targeting diverse antigens. Biosimilar, considered complex entities compared to small-molecule drugs, pose challenges in replication due to their biological nature. The manufacturing process involves rigorous comparability testing to ensure similarity in quality, safety, and efficacy with the reference product. Trastuzumab biosimilars, such as CT-P6, Ontruzant®, ABP 980, and PF-05280014, have shown efficacy in treating HER2-positive metastatic BCs, presenting a viable alternative to the reference product. The implications of monoclonal biosimilars extend beyond trastuzumab, with bevacizumab emerging as another significant biosimilar for BC treatment. The shift toward biosimilar aims to enhance accessibility to biologics by reducing costs. Health economic analyses indicate potential cost savings, contributing to the overall cost-effectiveness of biosimilar adoption. While concerns about switching between reference products and biosimilars exist, evidence suggests a lower risk of immunogenicity-related side effects with mAbs like trastuzumab. Monoclonal biosimilars present a promising avenue in BC therapy, demonstrating efficacy, safety, and potential cost savings. The integration of biosimilars into cancer treatment strategies offers a means to improve accessibility to effective care while addressing economic considerations in healthcare.

## Introduction and background

Breast cancer (BC) is a major global problem. In 2020, there were around two million women with BC. Reports from 2020 show that the total number of women who have survived BC for at least five years was found to be 7.8 million; thereby denoting it to be the most prevalent cancer in the world. The total disability-adjusted life years (DALYs) succumbed by women to BC was comparatively more on a global point than other types of cancers [1]. BC belongs to the heterogeneous disease group with multiple subtypes. Chemotherapy, genetic therapies, oncogene inactivation, tumor suppressor gene augmentation, cell-target suicide, virus-mediated oncolysis, immunomodulation (ectopic cytokine expression, immune enhancement), nanotechnology, and surgical procedures like breast-conserving surgery, lumpectomies, quadrant nephrectomies, and mastectomy are all available treatment options for treating BC. Recent advancements in science and technology have validated certain other concepts in the curative care of BC including monoclonal antibodies (mAbs) [2]. Antibodies prepared by cloning a unique B cell and binding them to the specific portions of an antigen are usually mAbs. They are similar to epitomes. In 1973, Schwaber discovered a number of techniques for manufacturing mAbs in an in vitro manner utilizing human-mouse hybrid cells [3,4]. These were used by Köhler and Milstein to excite the hybridomas produced by humans. This has led to the widespread manufacture of therapeutic antibodies [5,6]. Following this therapeutic alteration in cancer therapy, monoclonal antibiotic therapy has been put to use. Succeeding this development, there is an additional concept of using biologics in cancer therapy among patients with cancer. A regulatory body in Japan stated biosimilars as a biotechnology-derived product created to be comparable in terms of safety concerns, quality, and real-world effectiveness to an original biologic product of a different business that has previously received approval. Biosimilars are being manufactured in different places globally including Hyderabad and Mumbai in India. Biosimilars are highly approved biologics that hold a potential opportunity to improve the accessibility of biologics by reducing the cost burden. A biosimi­lar compound shares the same chemical composition as the approved medical compound although there are minimal differences owing to their production methodology and its complexities. Biosimilars could also be put through as biological products having a basic structure similar to the identified reference compound [7]. The biosimilarity development pathways involved applicable orthogonal analytical comparability methods, along with different clinical and non-clinical models. mAbs are seen to kill tumor cells in a varied manner including blocking ligand-receptor growth along with the survival pathways. The mechanism included antibody-dependent cellular cytotoxicity and complement-mediated cytotoxicity. The implications of these compounds have found a way to treat cancer in recent years although the research is nascent. Therefore, the present report aims to quote the treatment outcomes of monoclonal biosimilars in treating cancer and the advantage and cost-effectiveness of the same over other recently available treatment modalities [8]. This particular scientific literature piece highlights the therapeutic aspects of cancer therapy.

## Review

Methodology

As this is a narrative review, a comprehensive but strictly objective-driven search was conducted among the databases of Medline, Pubmed, Scopus, and Web of Science. We used the following keywords/search terms: "Monoclonal antibody," "biosimilar," "biosimilar in breast cancer," "breast cancer," and "biosimilar in cancer therapeutics." The articles published within the time frame of 2019 and 2023 were included in the present narrative review. A total of 18 articles were found, of which 16 were used for writing this narrative review.

Report

Biosimilars are complex entities compared to small-molecule drugs or generics. Biological medicines (biologics) are usually larger and manufactured using living organisms. Characterization of these biologics is difficult; therefore, it remains hard to replicate. In comparison to these biosimilars, it is important to understand the generics, which are usually small, identical molecules that have been synthesized chemically. Since 1985, there has been rigorous research in the field of medicine. Scientific reports have shown around 100 mAbs to be designated as drugs. Available mAbs target a large number of antigens and are found useful in treating immunologic diseases, reversing drug effects, and also in cancer therapy. mAb are homogenous preparations of identical antibodies. All antibodies have identical protein sequences thereby suggesting similar antigen recognition sites, affinity, biologic interactions, and downstream biologic effects [1,5,9].

Manufacturing of Antibodies and Advantages

Typically, a thorough comparability test between both the biosimilar and the mother product is necessary when creating biosimilar varieties from biological resources that have already received prior approval. The goal of the biosimilarity exercise is to preserve similarity in terms of quality, activity on a biological basis, safety concerns, and efficacy, while avoiding any clinically relevant alterations, in contrast to the reference product's marketing authorization application. Comparative nonclinical trials are conducted after a thorough physicochemical and biological investigation, which involves a comparison of quality characteristics [3,4]. To ascertain that the pharmacokinetics (PK), effectiveness, and safety of the product are equivalent to those of the reference product, clinical comparative testing is also necessary [3]. Creating biosimilars places more emphasis on data from the candidate's comprehensive physical, chemical, and biological presentations as well as on analytical tests in comparison to the reference product, when deciding whether to approve a novel biological rather than solely focusing on clinical trials [2,3,10]. After obtaining a mAb with the desired specificity, it must be manufactured in high numbers for therapeutic application. The first method of manufacturing was to construct a hybridoma (a cell-cell fusion) in which the antibody-producing cell was merged with an immortalized partner cell. A myeloma cell (a malignant B cell) is commonly utilized as a companion because it may multiply continuously in vitro. The clinical package is expected to include a phase 1 and phase 3 trial to confirm the reference product's approval. Clinical biosimilarity could be established by three confirmatory PK along with other pharmacodynamic (PD) investigations [11]. The European Medicines Agency (EMA) has produced various guidance documents to help sponsors develop biosimilars, which includes a guideline for biosimilar mAbs that is specific to the product [12,13]. Directing and evaluating a biosimilar's comparability is done using a case-to-case approach by EMA. Clinical progress of trastuzumab biosimilars is covered with reference to EMA recommendations in this article; there are some slight variations with FDA guidelines, but they are minimal. Furthermore, the biologics production process is more complicated than that of small-molecule pharmaceuticals, requiring different cloning stages. The process also includes selecting, followed by maintaining, and increasing the cell line. This was accompanied by isolating and purifying. Characterizing the result was the concluding step. Small-molecule medications are made using a series of predictable chemical events that can be consistently duplicated to make identical duplicates. Furthermore, the biologics production process is more complicated than that of small-molecule pharmaceuticals, requiring different cloning stages, in selecting, maintaining, and also increasing the cell line, as well as isolating, purifying, and characterizing the result. Small-molecule medications are made using a series of predictable chemical events that can be consistently duplicated to make identical duplicates [14].

Effectiveness

Trastuzumab is available as biosimilars under the brand names CT-P6 (Herzuma®; Korean Republic), Ontruzant®; Samsung Bioepis, ABP 980 (Kanjinti®), and PF-05280014 (Trazimera®) [9-13]. As with Herceptin®, the reference product, CT-P6 binds to the same HER2 epitope with great affinity and specificity (Genentech) [9,10]. In 2018, the Government Agency of Medicine in Europe and the US Food and Drug Administration approved CT-P6 to treat HER2-positive metastatic BC at their early stage [14-16]. Clinical trials are supplemented by real-world studies showing the long-term safety and efficacy of medications among a wider patient population, as well as in various settings and in conjunction with other treatments. For instance, as part of dual HER2-targeted therapy, reference trastuzumab (RTZ) and pertuzumab (Perjeta®; Genentech) are increasingly combined. Trastuzumab targets a separate location of the HER2 receptor than the mAb pertuzumab, giving it a complementary pattern of action. The combination of trastuzumab along with chemotherapy has been found to be clinically effective as it acts by dual HER2-targeting with trastuzumab and pertuzumab. This was found to have improved clinical outcomes in HER2-positive metastatic breast tumors in their early stage [9-12].

Implications

Trastuzumab is the mAb that has the most impact in the treatment of HER2 disease because it has the ability to shift the disease's natural course. HER2 amplification is seen in HER2-positive BC and metastatic gastric cancer [1]. Together with bevacizumab (a mAb that binds vascular endothelial growth factor (VEGF)) is a biosimilar to trastuzumab. In Europe, VEGF is approved in combination with other drugs to prevent tumor angiogenesis. Adult patients with metastatic BC should be treated first with paclitaxel [3]. Bevacizumab's approval as a biosimilar is a crucial step toward ensuring that more cancer patients receive mAbs as part of their treatment, but it is crucial to remember that this drug is only authorized for the BC treatment in Europe and is not identified among the FDA's approved indications for this disease.

Switching of Trastuzumab: Mother Product vs. Biosimilar Version

Biosimilars mimicking Trastuzumab, that have received approval, work similarly to the original medication. However, there have been some lingering questions about switching between a reference product with its biosimilar. Due to exposure to possibly different distinct combinations of epitopes resulting from minute variations between the reference product and the biosimilar, switching could enhance immunogenicity. Growing data supports the use of biosimilar versions of many drugs, including etanercept, infliximab, and adalimumab, in phase 3 trials that examine the effects of switching. Since trastuzumab is a mAb with limited immunogenic potential, there is a lower chance of immunogenicity-related side effects. However, switching is less frequent than for conditions requiring long-term biological treatment. In situations of early BC, trastuzumab is used for a year; in cases of metastatic BC, trastuzumab is given until the illness advances [10]. Given the brief duration of the therapy, a cost-benefit analysis might be used to compare switching to a more cost-effective version with the implementation costs associated with the switch.

Health Economics

Biosimilars were created with the intention of lowering medical costs. Biosimilars have a typical starting price that is 20% to 30% lower than the reference products. They also provide competition to previously exclusive areas along with additional confidential benefits [8]. The CT-P6 treatments in the neoadjuvant scenery were approximately around €1500 less expensive than RTZ in a study. Although this may appear to be a small saving, it should be recognized that the frequency of neoadjuvant cycles needed is less than in the other contexts. With an average follow-up length of eight years, the CLEOPATRA experiment was updated in 2020. Until now two pharmacoeconomic studies based on the most recent data from the CLEOPATRA experiment have been published. Moriwaki et al. used a partitioned survival model to assess the cost-effectiveness of HER2-positive metastatic BC-targeted therapy from the perspective of Japanese public healthcare payers [8]. With an ICER value of 183, 901 USD/QALY, the pertuzumab group gained 0.949 QALYs. In addition, Cheng, et al. concluded that the pertuzumab group gained 0.73 more QALYs, and had an ICER value of 272,244 USD/QALY [16].

## Conclusions

A number of cancers have benefited from the use of new diagnostic methods, such as molecular profiling, which have improved clinical outcomes, including overall survival. Contrarily, the high cost of biologic drugs can make it impossible for patients to have the best care feasible. The introduction of cost-effective biosimilar drugs into the therapeutic arsenal increases the potential to reduce healthcare costs while also improving the accessibility of effective cancer cures. Oncology biosimilars have been established with better efficacy and safety based on previous literature reports of clinical trials and quality physicochemical data along with real-world settings.
